# PDBench: evaluating computational methods for protein-sequence design

**DOI:** 10.1093/bioinformatics/btad027

**Published:** 2023-01-13

**Authors:** Leonardo V Castorina, Rokas Petrenas, Kartic Subr, Christopher W Wood

**Affiliations:** School of Informatics, University of Edinburgh, 10 Crichton Street, Newington, Edinburgh EH8 9AB, UK; School of Biological Sciences, University of Edinburgh, Roger Land Building, Edinburgh EH9 3FF, UK; School of Informatics, University of Edinburgh, 10 Crichton Street, Newington, Edinburgh EH8 9AB, UK; School of Biological Sciences, University of Edinburgh, Roger Land Building, Edinburgh EH9 3FF, UK

## Abstract

**Summary:**

Ever increasing amounts of protein structure data, combined with advances in machine learning, have led to the rapid proliferation of methods available for protein-sequence design. In order to utilize a design method effectively, it is important to understand the nuances of its performance and how it varies by design target. Here, we present PDBench, a set of proteins and a number of standard tests for assessing the performance of sequence-design methods. PDBench aims to maximize the structural diversity of the benchmark, compared with previous benchmarking sets, in order to provide useful biological insight into the behaviour of sequence-design methods, which is essential for evaluating their performance and practical utility. We believe that these tools are useful for guiding the development of novel sequence design algorithms and will enable users to choose a method that best suits their design target.

**Availability and implementation:**

https://github.com/wells-wood-research/PDBench

**Supplementary information:**

Supplementary data are available at *Bioinformatics* online.

## 1 Introduction

The goal of protein design is to create novel amino acid sequences with useful properties and functions. An important part of this process is determining sequences that will fold to a target structure, and this can be thought of as the ‘inverse protein folding problem’ (Yue and Dill, 1992). To address this challenge, many successful approaches for designing proteins have been developed, but computational protein design (CPD) has quickly become the standard approach (Woolfson, 2021).

Current methods for benchmarking protein design methods focus on sequence recovery, where the backbones of natural proteins with known amino-acid sequences are passed as the input and the accuracy of the method is measured by identity between the predicted sequence and the true sequence (Qi and Zhang, 2020; Strokach *et al.*, 2020; Zhang *et al.*, 2020). However, accuracy values do not capture the real-world utility of a design method. Ultimately, *we must move beyond simplistic methods for evaluating design methodologies* and provide information to users that will help them to assess whether a specific method will be appropriate for their target application.

Here, we describe PDBench, a set of protein structures and associated tools for benchmarking the performance of CPD methods. PDBench generates a rich set of metrics to give a more holistic view of performance.

## 2 Materials and methods

Our benchmark set contains 595 protein structures spanning 40 protein architectures that are clustered into 4-fold classes: mainly-*α*, mainly-*β*, *α*–*β* and special, as presented in the CATH database (Knudsen and Wiuf, 2010). Crystal structures with maximum resolution of 3 Å were chosen to cover the structural diversity present in the PDB (see 1). This ensures that the performance is evaluated on high- and low-quality inputs (see Supplementary Fig. S2) and the results are not biased towards the most common protein architectures.

**Fig. 1.**
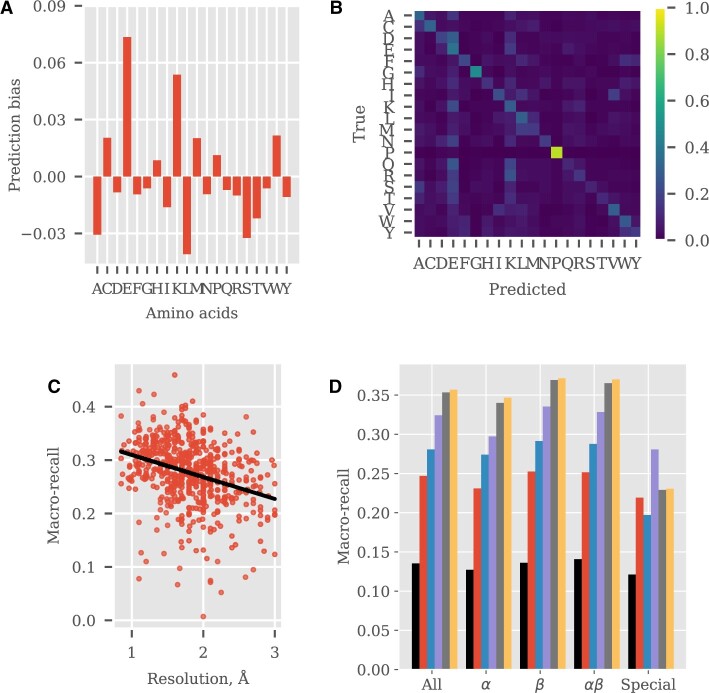
Example plots produced by PDBench. (**A**–**C**) Selected performance plots for ProDCoNN. (**A**) Prediction bias of predictions relative to abundance of amino acids in the dataset. The model has a negative bias towards common residues such as leucine and alanine, indicating that there is not a bias for the most common class. (**B**) Correlation between performance and resolution of protein structures. (**C**) Prediction confusion matrix between predicted residue and real residue in the sequence. (**D**) Performance comparison plot between all models tested across different types of folds (bars in group from left to right): ProteinSolver (black), EvoEF2 (red), ProDCoNN (blue), Rosetta (purple), DenseCPD (gray) and DenseNet (yellow), see also Supplementary Figures S7 and S8 (A color version of this figure appears in the online version of this article)

Benchmarking tool: We have developed an open-source benchmarking library, written in Python (https://github.com/wells-wood-research/PDBench). The user supplies PDBench with a prediction matrix (in .csv format) and a dataset map (in .txt format), and it generates metrics for each model in a plot, as well as the option to generate comparison plots between different models to compare their performance. The software is not limited to the benchmarking set we have created, the user can specify any set of structures and the AMPAL library will be used to read the protein sequences and optionally replace non-canonical residues with standard amino acids (Wood *et al.*, 2017). The programme DSSP is used to assign the secondary structure for each residue (Joosten *et al.*, 2011). The CATH database (Knudsen and Wiuf, 2010) is used to assign protein architecture. Figure 1 shows a sample output for several models.

Metrics: We calculate four groups of metrics: (1) recall, precision, AUC, F1 score, Shannon’s entropy and prediction bias *for each amino acid class*; (2) accuracy, macro-precision, macro-recall, similarity and top-3 accuracy *for each protein chain*; (3) accuracy, macro-precision, macro-recall, similarity and top-3 accuracy *for each secondary structure type* and (4) accuracy, macro-precision, macro-recall, similarity and top-3 accuracy *for each protein architecture*. As shown in Supplementary Figure S2 (right), the numbers of amino acids in proteins are heavily imbalanced, meaning that a model overpredicting the most common amino acid may obtain an accuracy higher than random. Macro-recall is an accuracy score resistant to class imbalance which allows a fairer comparison of models.

Prediction bias is a metric measuring the discrepancy between the occurrence of a residue and the number of times it is predicted (see Fig. 1A and Supplementary Fig. S5). To account for functional redundancy between amino acids, the relative frequency of substitution of amino acids in nature is used to calculate a similarity score (Henikoff and Henikoff, 1992). PDBench also outputs torsion angle comparison plots between true and predicted residues, if structural models are provided, which is useful to further explore overprediction (see Fig. 1B and Supplementary Fig. S4).

Models evaluated: We tested two state-of-the-art physics-based methods: ‘EvoEF2’ (Huang *et al.*, 2019b) and ‘Rosetta’ (Das and Baker, 2008). We also tested deep-learning methods: ‘ProDCoNN’ (Zhang *et al.*, 2020) (CNN), ‘DenseCPD’ (Huang *et al.*, 2019a) (CNN), ‘DenseNet’ (Huang *et al.*, 2019a) (CNN) and ‘ProteinSolver’ (Strokach *et al.*, 2020) (GNN). Code for ProDCoNN, DenseCPD and DenseNet was not available, so we re-implemented them using Keras and filtering out the benchmark structures from the training set (Supplementary Fig. S6).

## 3 Discussion

**Fig. 2.**
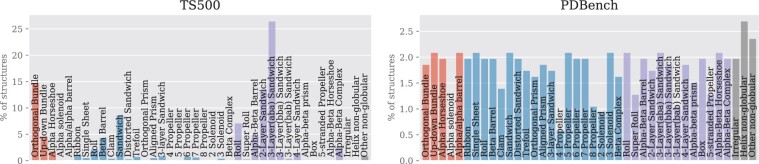
Comparison between the TS500 benchmark (O’Connell *et al.*, 2018), commonly used in the literature, (left) and our fold-balanced benchmark PDBench (right). The PDBench benchmark comprises 40 protein architectures grouped into 4 categories: mainly-*α* (red)—70 chains, mainly-*β* (blue)—282 chains, *α*–*β* (purple)—196 chains and special (yellow)—47 chains (A color version of this figure appears in the online version of this article)

As an example, we used PDBench to compare a range of published methods for sequence design (see Supplementary Fig. S3). We divided our benchmark set (595 structures) into four categories of protein folds, each with a balanced proportion of structures for each category as shown in (Fig. 2, right), unlike other benchmarks such as TS500, which is heavily unbalanced (Fig. 2, left). When considering accuracy metrics along with similarity to the target structures (Supplementary Fig. S3), there is a marked difference in the performance of all the design algorithms across the different fold classes. It is interesting that all of the deep-learning-based methods performed well when designing ‘mainly *β*’ structures, as these are challenging design targets (Huang *et al.*, 2016; Woolfson *et al.*, 2015). Furthermore, the accuracy of sequence recovery was more strongly correlated with resolution in the *β*-containing classes (Supplementary Fig. S3), suggesting that the sequence preferences in *β* structure are closely linked to subtle details in the backbone conformation. The performance of the ProteinSolver was lower than expected, given the reported performance (Strokach *et al.*, 2020). We believe that this is due to leakage of information regarding side chains identities, if they are provided in the input model (Supplementary Section S4). As a result, while the method might be suitable for protein engineering, it is ill suited to *de novo* design where only backbone atoms are provided as an input.

While sequence recovery is an important metric in understanding the performance of a sequence design method, it is not sufficient to fully understand its properties. Furthermore, a static, single structure view of a protein is not representative of the behaviour of a protein in solution. The ultimate test is to produce design in the lab, but further computational analysis of the models can also generate useful information on designs (Goldenzweig *et al.*, 2016; Ludwiczak *et al.*, 2018; Ollikainen and Kortemme, 2013; Stam and Wood, 2021).

Our design-method agnostic benchmark and tools aim to shed light on the behaviour of CPD algorithms. We believe that this information will be of use to developers of CPD algorithms, especially when combined with modern methods of structure prediction (Chowdhury *et al.*, 2021; Jumper *et al.*, 2021; Rives *et al.*, 2019; Wu *et al.*, 2022). It also provides users of these methods crucial information regarding the appropriateness of the design method to their application.

## Supplementary Material

btad027_Supplementary_Data

## Data Availability

The data underlying this article are available in through the Protein Data Bank (PDB), which can be accessed here: http://www.wwpdb.org. Source code is available on GitHub: https://github.com/wells-wood-research/PDBench.
