# Supplementary material for: PDBench: evaluating computational methods for protein-sequence design
Source: Bioinformatics. 2023 Jan 13;39(1):btad027. doi: 10.1093/bioinformatics/btad027 (PMC9869650; doi:10.1093/bioinformatics/btad027)
Supplement: btad027_Supplementary_Data [file btad027_supplementary_data.pdf]

PDBench: Evaluating Computational Methods for Protein Sequence Design

Supplementary Materials

1 Benchmark Details

1.1 TS500

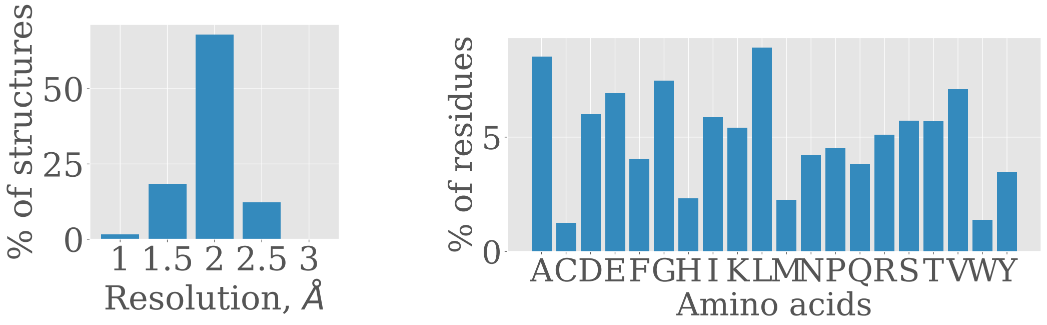

Figure 1: Resolution and amino acid distribution in the TS500 set

1.2 PDBench (ours)

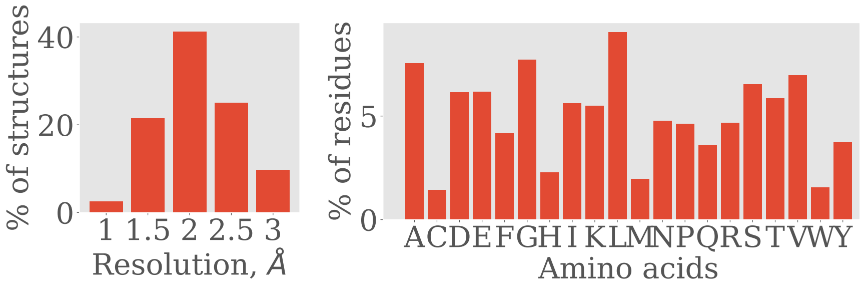

Figure 2: Resolution and amino acid distribution in the PDBench set.

2 Benchmark Results

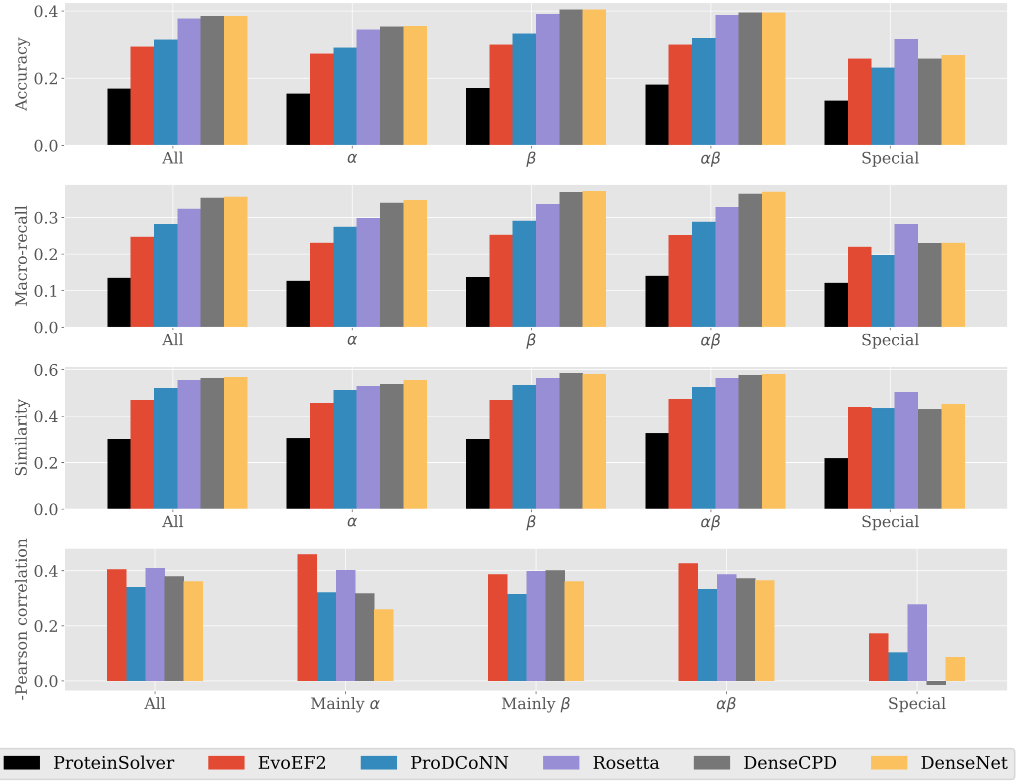

Figure 3: The first two plots compare performances of models across classes of folds. The plot at the bottom shows the negated correlation coefficient (Y axis) between macro-recall and the resolution (in Å) of the input structure. All p-values were significant ($< 10^{-8}$) except for ProteinSolver (0.3) which is excluded from the plot.

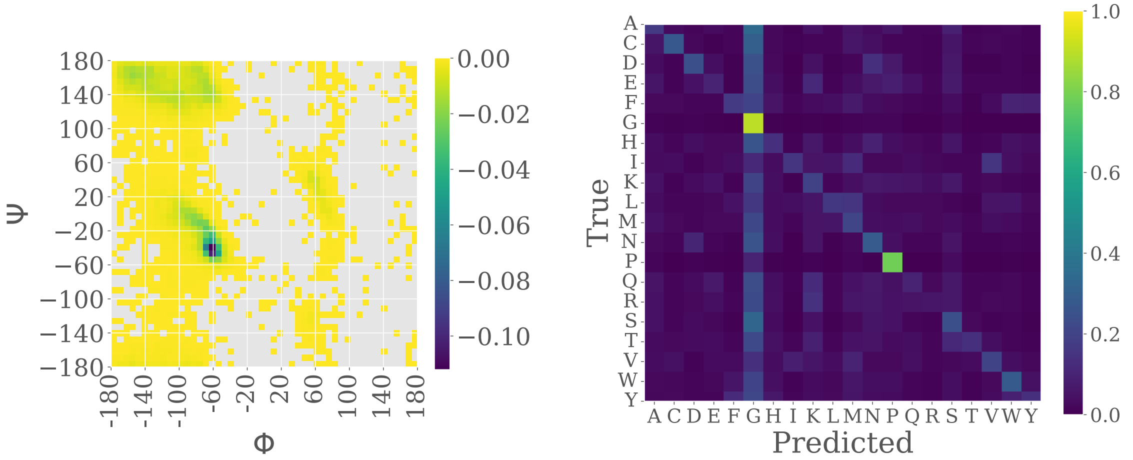

Figure 4: Φ and Ψ plots to explore Glycine overprediction. Left: A torsion angle plot showing normalized frequency difference between true and predicted number of glycine amino acids. Negative values indicate increased glycine frequency in predicted sequences. Right: A confusion matrix showing the confusion frequency for amino acid pairs.

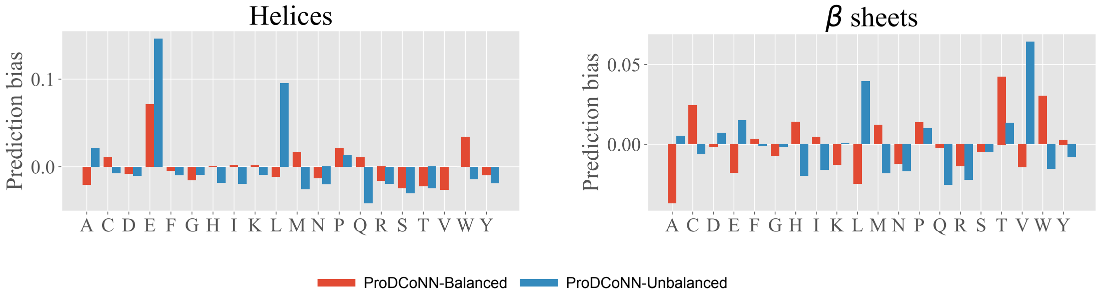

Figure 5: Prediction bias comparison for ProDCoNN-Balanced (red) and ProDCoNN-Unbalanced models across all the structures in the benchmark and for each type of residue. The left plot represents bias on α-helical structures, while the plot on the right is for β-sheets. Prediction bias is calculated as deviation of the predictions from the real frequency of residues in the benchmark structures.

3 Input Data Pipeline

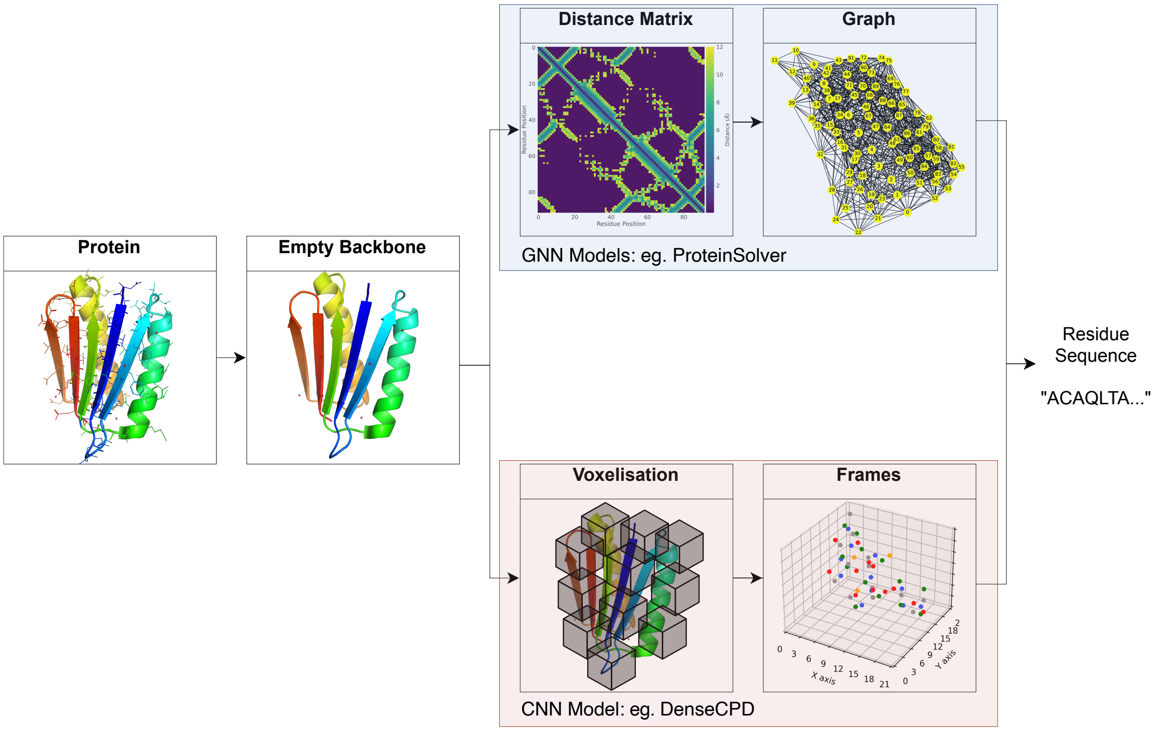

Figure 6: Illustration of the data pipeline. High-quality 3D structures of proteins are obtained from a database. The side-chains of each residues are removed so to produce an empty backbone. The GNN models calculate the distances between each residue in the protein to produce a distance matrix which is used for the production of a graph. The CNN model, on the other hand, voxelises areas of space ("frame") around each residue, with the Cα at the center of it. Both models predict the identity of the side-chains of the residues giving a sequence of predicted residues to obtain the input 3D structure.

4 ProteinSolver Distance Matrix with and without Poly-Glycine Input

Protein Solver Distance Matrices using 1QYS protein with and without sidechain atoms (labels). ProteinSolver claims to use the heaviest atom in the residue and as confirmed by private conversation received on June 5th at 20:59, they "include both backbone **and side chain atoms** when calculating nearest distances".

The side chains atoms determine the identify of the amino acids and are therefore labels. In a truly *de novo* design setting, only the backbone structure (without side chains) would be available.

The macro-recall performance dropped from 36.6 to 13.3 when using an empty backbone (poly-glycine) input. The performance drop is only observed for ProteinSolver while it remains constant for all other models.

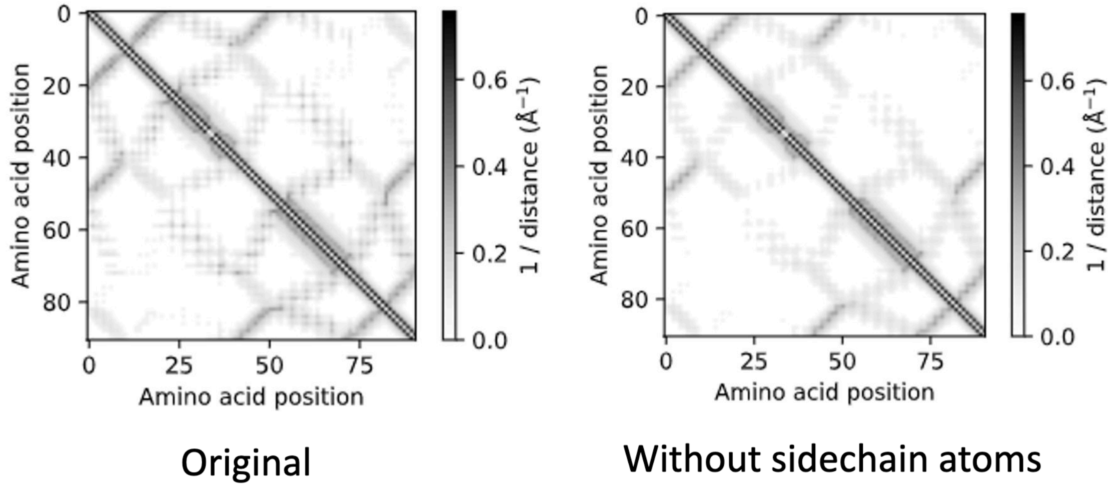

Figure 7: The distance matrix changes if side-chains (labels) are included in the .pdb input. The two distance matrices should be identical as only the empty backbone atoms should be used, as they are the only ones available in *de novo* settings, meaning it probably presents label leakage.

5 Physics Models Commands

We evaluated two state-of-the-art physics-based methods: **EvoEF2** (Huang, Pearce, and Zhang 2019) and **Rosetta** (Alford et al. 2017). We used the following commands to run fixed backbone design protocols:

EvoEF2: ./EvoEF2 –command=ProteinDesign –ppint –design_chains=B –pdb=structure.pdb to design chain B from structure.pdb.

Rosetta (version 3.12): ./fixbb.static.linuxgccrelease -s structure.pdb -linmem_ig 10 -ignore_unrecognized_res -resfile file.txt. Resfile was used to select specified chain.

6 Benchmark structures

Our benchmark set contains 595 protein structures spanning 40 protein architectures.

PDB code + chain: 1xg0C, 3g3zA, 3rf0A, 4i5jA, 2ptrA, 3f0cA, 4a5uB, 2p57A, 2q0oC, 6er6A, 1h32A, 3e3vA, 3cxbA, 1dvoA, 5dicA, 2bnmA, 4pfoA, 2ebfX, 3giaA, 1a41A, 3cexA, 4ebbA, 3jrtA, 3wfdB, 4v1gA, 3qb9A, 3abhA, 3nvoA, 2o1kA, 5x56A, 2ra1A, 4adzA, 2p6vA, 3k4iA, 4lctA, 4adyA, 4zhbA, 4p6zG, 4nq0A, 3dadA, 2vq2A, 4dloA, 2of3A, 4y5jA, 2pm7A, 2hr2A, 3ro3A, 3bqoA, 3ut4A, 2yhcA, 4k6jA, 3iisM, 5agdA, 2fbaA, 3e7jA, 1v7wA, 3a0oA, 4wu0A, 4ozwA, 4cj0A, 1gxmA, 5m7yA, 4fnvA, 5gzkA, 4ayoA, 3wkgA, 3vsnA, 2jg0A, 4j5tA, 4ktpA, 4mqwA, 5lf2A, 5mriA, 5ol4B, 1bx7A, 3ca7A, 3tvjA, 3tbdA, 1uzkA, 5bq8A, 3klkA, 1b8kA, 1v6pA, 4hquA, 4k8wA, 6a2qA, 1lpbA, 3hrzB, 6fmeB, 2aydA, 2ra8A, 4fzqA, 3d4uB, 3wwlA, 2r01A, 1lslA, 3f3fC, 2q4zA, 2de6A, 3d9xA, 2hjeA, 3mcbB, 2y8nB, 3witA, 1ya5T, 2dyiA, 3kyfA, 2v76A, 2e12A, 1g3pA, 4o06A, 3fb9A, 2p38A, 1igqA, 4hhvA, 3teeA, 5j3tA, 5h3xA, 3zbdA, 5d7uA, 5zcjC, 5u1mA, 1wthD, 4rg1A, 1kt6A, 2ja9A, 1i4uA, 4i86A, 1o7iA, 1x8qA, 2ichA, 3dzmA, 3n91A, 1luzA, 4lqzA, 4i1kA, 5xlyB, 3a35A, 3tdqA, 4mxtA, 3wjtA, 3buuA, 3ksnA, 2w7qA, 2yzyA, 4z48A, 3bk5A, 4qa8A, 2byoA, 3bmzA, 4egdA, 4joxA, 3h6jA, 2bhuA, 1pmhX, 6ggrA, 4dqaA, 4v2bA, 4weeA, 2w07B, 4r9pA, 2r2cA, 2r0hA, 4aqoA, 4luqC, 3iagC, 1k5nA, 2ygnA, 3bwzA, 4fmrA, 1njhA, 4hi6A, 1pkhA, 1gp0A, 3q1nA, 2ag4A, 2v3iA, 3ty1A, 1gprA, 3aihA, 4c4aA, 1tulA, 4a02A, 4c08A, 4maiA, 1jovA, 3wmvA, 2fdbM, 1dqgA, 1xzzA, 6i18A, 4i4oA, 4efpA, 5yh4A, 3h6qA, 5bowA, 5vi4A, 2vxtI, 3vwcA, 4lo0C, 1sr4C, 2dpfA, 3dzwA, 3a0eA, 1xd5A, 4h3oA, 4tkcA, 5j76A, 3mezC, 4gc1A, 1b2pA, 4le7A, 4oitA, 6b0gE, 1z1yB, 1vmoA, 2gudA, 4r6rE, 5krpC, 5v6fA, 4pitA, 6flwA, 4ddnD, 3apaA, 5gvyA, 1c3mA, 4mq0A, 3wocA, 3aqgA, 3towA, 2qp2A, 1nykA, 2bmoA, 2gbwA, 1rfsA, 4aivA, 3gkeA, 2nwfA, 1jm1A, 2qpzA, 5cxmB, 3dqyA, 3d89A, 2b1xA, 4qdcA, 2q3wA, 3c7xA, 1genA, 1itvA, 3s18A, 4rt6B, 3cu9A, 3wasA, 6ms3B, 6frwA, 3k1uA, 5aycA, 5c0pA, 4n1iA, 3r4zA, 1tl2A, 4u6dA, 1oygA, 4qqsA, 3qz4A, 5a8cA, 4pvaA, 3kstA, 5flwA, 6gy5A, 1cruA, 1suuA, 3o4pA, 2p4oA, 4mzaA, 5gtqA, 3dr2A, 3dasA, 3g4eA, 2fp8A, 5hx0B, 1npeA, 1s1dA, 2zwaA, 3a72A, 2zb6A, 3scyA, 3b7fA, 3al9A, 3o4hA, 4pxwA, 4wk0A, 2w18A, 5em2A, 1sq9A, 1xipA, 4h5iA, 1jofA, 5ic7A, 5k19A, 6e1zA, 2z2nA, 6e4lA, 6fkwA, 6damA, 1flgA, 4cvbA, 4mh1A, 1z68A, 2z3zA, 4q1vA, 1xfdA, 5d7wA, 1kapP, 3laaA, 1p9hA, 3ultA, 3s6lA, 2xqhA, 4dt5A, 5m5zA, 5lw3A, 1k5cA, 1k4zA, 2ntpA, 3bh7B, 2j8kA, 2vfoA, 3n6zA, 1hf2A, 2x3hB, 1rmgA, 6mfkA, 1l0sA, 2xt2A, 5nzgA, 3kweA, 2w7zA, 1lktA, 3facA, 3pyiB, 2casA, 1gppA, 3maoA, 1ut7A, 1hxrA, 1t61A, 4qjvA, 3lywA, 3dalA, 5hqhA, 3u7zA, 3r90A, 1tp6A, 3s9xA, 2ex5A, 3gbyA, 5kvbA, 2cu3A, 1c1yB, 5f6rA, 4a6qA, 2w56A, 4lqbA, 4oobA, 3oajA, 3n8bA, 3jumA, 2prxA, 5b1rA, 1ewfA, 4m4dA, 2obdA, 6baqA, 1usuB, 3e8tA, 3aotA, 2rckA, 3l6iA, 3uv1A, 3bqwA, 5mprA, 1kkoA, 4cd8A, 1vd6A, 2g0wA, 4lanA, 3s83A, 2v3gA, 3fkrA, 4z0gA, 3sggA, 5zjbA, 2xfrA, 4g8tA, 5n6fA, 1muwA, 2qhqA, 3h35A, 3kluA, 3fn2A, 2od6A, 1kcfA, 3nlcA, 2zw2A, 4ftxA, 3u2aA, 2hiqA, 1xkpC, 6ih0A, 5c12A, 1w4rA, 3c0fB, 3nbmA, 2r6zA, 5hxdA, 1chdA, 3do8A, 3gohA, 1n0eA, 2q82A, 5kxhA, 3oqiA, 2x4lA, 3d3kA, 3l46A, 2fkcA, 5jphA, 3nytA, 3rhtA, 3dkrA, 2psbA, 1tc5A, 3vrdB, 2je3A, 3g5sA, 1jkeA, 4at0A, 1vi4A, 4u8pC, 4ntcA, 5ipyA, 5nakA, 4z24B, 4opcA, 5cdkA, 2b0aA, 4n2pA, 1j5uA, 1vzyB, 1vq0A, 4ipuA, 4dq9A, 4jtmA, 3gs9A, 3adyA, 3mi0A, 3ib7A, 3g91A, 1vr7A, 4zx2A, 1ds1A, 3zwfA, 1hq0A, 3hbcA, 3p8kA, 1wraA, 3t91A, 3c9fA, 2imhA, 1um0A, 5y0mA, 5u4hA, 3zh4A, 3swgA, 5ujsA, 3nvsA, 2o0bA, 2pqcA, 3slhA, 1rf6A, 4n3pA, 5bufA, 3rmtA, 4fqdA, 1ud9A, 1t6lA, 1rwzA, 3ifvA, 1iz5A, 3lx2A, 1u7bA, 5tupA, 5h0tA, 5v7mA, 3fdsC, 3aizA, 1b77A, 3p91A, 1dmlA, 3hslX, 2z0lA, 6nibA, 2jerA, 1xknA, 1zbrA, 3hvmA, 1jdwA, 5wpiA, 1g61A, 1h70A, 5m3qA, 1ynfA, 3wn4A, 1io0A, 4rcaB, 4fcgA, 4ecoA, 3wpcA, 4im6A, 4cnmA, 5hzlB, 4fs7A, 2xwtC, 3e4gA, 4wp6A, 5il7A, 1z7xW, 4u7lA, 6fg8A, 2wfhA, 2fy7A, 5wwdA, 1j3aA, 1omzA, 3emfA, 1xw3A, 3h4rA, 3essA, 1o22A, 4ktbA, 1jh6A, 3n08A, 5tsqA, 3e9vA, 4j7hA, 1i4jA, 2wnfA, 3v1aA, 3coqA, 2f60K, 4zgmA, 1i7wB, 6g6kA, 1pbyC, 1a92A, 3alrA, 2wjvD, 2a26A, 1devB, 4l0nA, 4ayaA, 3zxcA, 4pkfB, 2b1yA, 4dncD, 4jpnA, 4e18B, 3vepX, 3v4yB, 1xawA, 1ykhA, 2p64A, 6bscB, 2z3xA, 4uzzB, 3thfA, 1wq6A, 4ke2A, 4lhfA, 2v66B, 3lczA, 2h4oA, 4wjwA, 3kvpA, 3e56A, 3bk3C, 2ds5A, 3zoqB, 3nfgB, 4ksnA, 3ua0A, 3nrtA, 4a9aC, 6hikL

7 DenseCPD Architecture (claimed to be DenseNet)

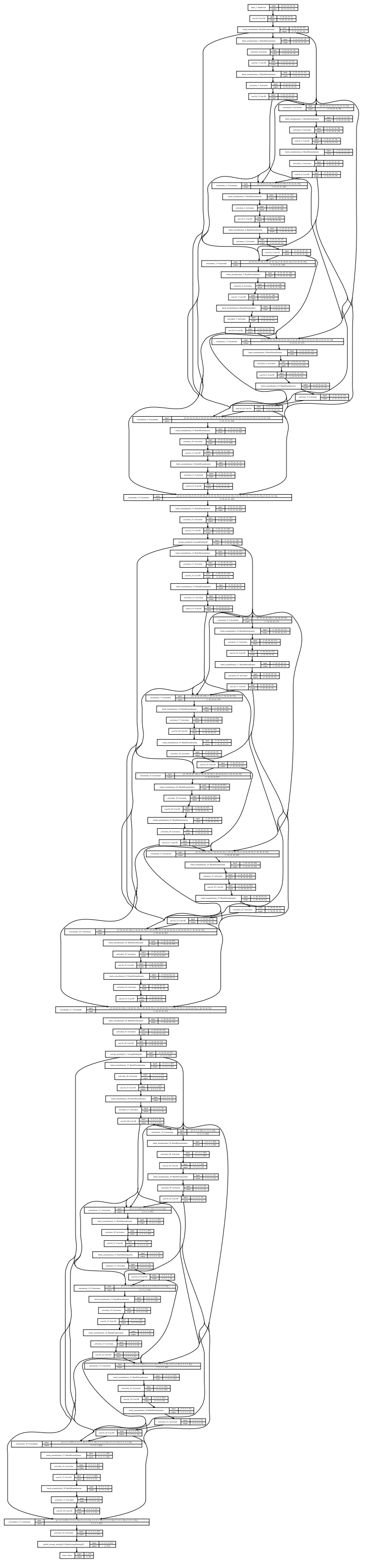

Figure 8: Implementation of ”DenseNet” architecture based on the DenseCPD from the paper figure.

8 DenseCPD Architecture (actual)

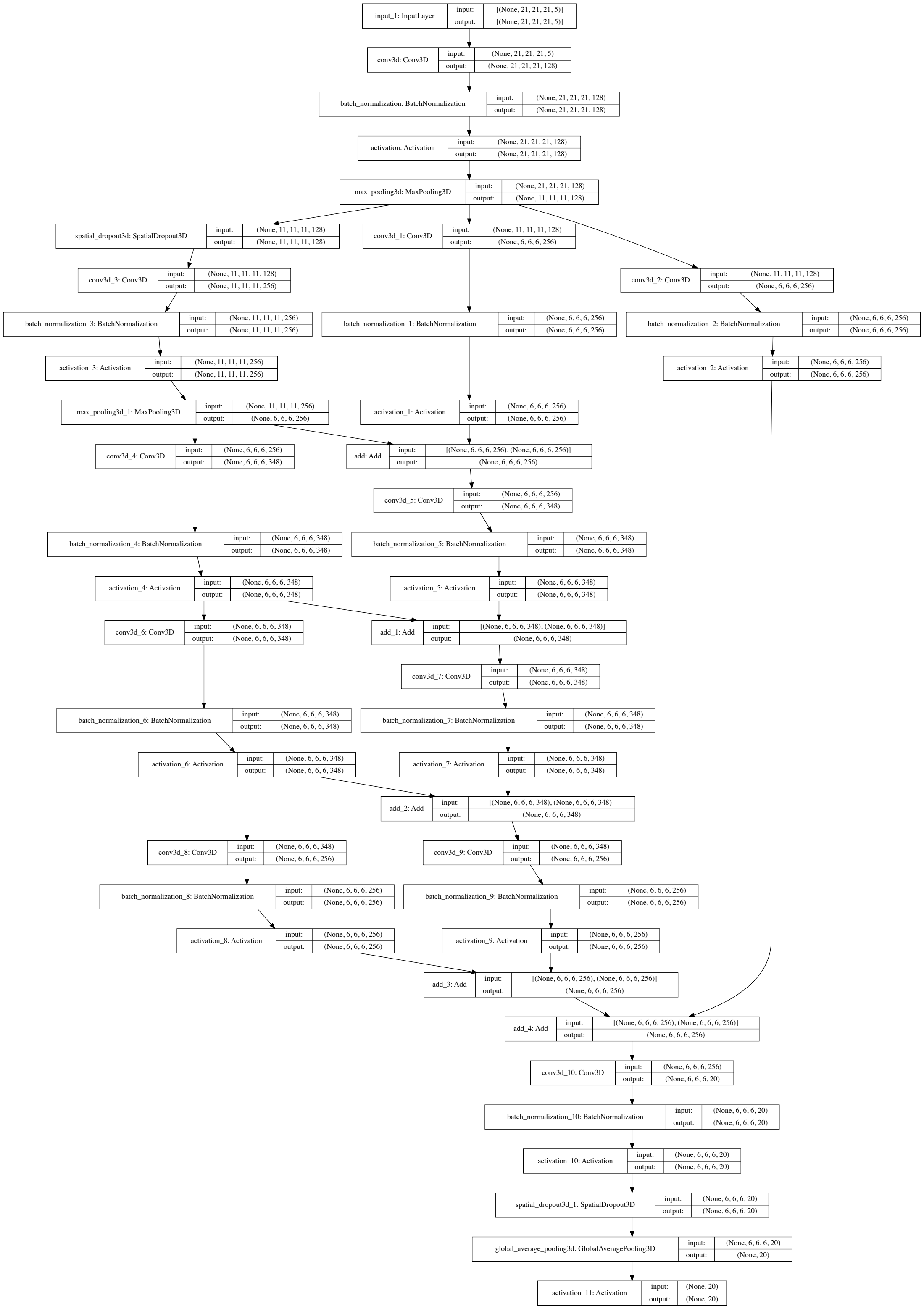

Figure 9: Architecture of the DenseCPD obtained from the model.json file from the authors.

References

Alford, R. F.; Leaver-Fay, A.; Jeliazkov, J. R.; O'Meara, M. J.; DiMaio, F. P.; Park, H.; Shapovalov, M. V.; Renfrew, P. D.; Mulligan, V. K.; Kappel, K.; Labonte, J. W.; Pacella, M. S.; Bonneau, R.; Bradley, P.; Dunbrack, R. L.; Das, R.; Baker, D.; Kuhlman, B.; Kortemme, T.; and Gray, J. J. 2017. The Rosetta All-Atom Energy Function for Macromolecular Modeling and Design. *Journal of Chemical Theory and Computation*, 13(6): 3031–3048.

Huang, X.; Pearce, R.; and Zhang, Y. 2019. EvoEF2: accurate and fast energy function for computational protein design. *Bioinformatics*, 36(4): 1135–1142.
